# Study of the Metal–Support Interaction and Electronic Effect Induced by Calcination Temperature Regulation and Their Effect on the Catalytic Performance of Glycerol Steam Reforming for Hydrogen Production

**DOI:** 10.3390/nano11113149

**Published:** 2021-11-22

**Authors:** Songshan Zhu, Yunzhu Wang, Jichang Lu, Huihui Lu, Sufang He, Di Song, Yongming Luo, Jiangping Liu

**Affiliations:** 1 Faculty of Environmental Science and Engineering, Kunming University of Science and Technology, Kunming 650500, China; Zhuss525@163.com (S.Z.); wyzkust@163.com (Y.W.); lujichangc7@kust.edu.cn (J.L.); luhuihuiluhuihui@163.com (H.L.); songdi0512@163.com (D.S.); environcatalysis@kust.edu.cn (Y.L.); 2 The Innovation Team for Volatile Organic Compounds Pollutants Control and Resource Utilization of Yunnan Province, Kunming 650500, China; 3 The Higher Educational Key Laboratory for Odorous Volatile Organic Compounds Pollutants Control of Yunnan Province, Kunming 650500, China; 4 Research Center for Analysis and Measurement, Kunming University of Science and Technology, Kunming 650093, China; 5 Faculty of Chemical Engineering, Kunming University of Science and Technology, Kunming 650500, China

**Keywords:** hydrogen production, glycerol steam reforming, nickel-based supported catalyst, electronic and metal–support interactions

## Abstract

Steam reforming of glycerol to produce hydrogen is considered to be the very promising strategy to generate clean and renewable energy. The incipient-wetness impregnation method was used to load Ni on the reducible carrier TiO_2_ (P25). In the process of catalyst preparation, the interaction and electronic effect between metal Ni and support TiO_2_ were adjusted by changing the calcination temperature, and then the activity and hydrogen production of glycerol steam reforming reaction (GSR) was explored. A series of modern characterizations including XRD, UV-vis DRS, BET, XPS, NH_3_-TPD, H_2_-TPR, TG, and Raman have been applied to systematically characterize the catalysts. The characterization results showed that the calcination temperature can contribute to varying degrees of influences on the acidity and basicity of the Ni/TiO_2_ catalyst, the specific surface area, together with the interaction force between Ni and the support. When the Ni/TiO_2_ catalyst was calcined at 600 °C, the Ni species can be produced in the form of granular NiTiO_3_ spinel. Consequently, due to the moderate metal–support interaction and electronic activity formed between the Ni species and the reducible support TiO_2_ in the NiO/Ti-600C catalyst, the granular NiTiO_3_ spinel can be reduced to a smaller Ni^0^ at a lower temperature, and thus to exhibit the best catalytic performance.

## 1. Introduction

Hydrogen energy has gradually attracted people’s research interest because it is regarded as a promising high-efficiency clean energy and is widely used in the chemical and petroleum industries, especially in fuel cell power generation [1,2]. However, the current primary source of hydrogen production is from fossil energy reforming, contributing to a large amount of carbon dioxide (CO_2_) gas emission as well; for example, producing 1 ton of hydrogen from shale gas will release about 10 tons of CO_2_ gas [3]. Furthermore, the physical state of hydrogen makes its storage and transportation difficult, which is not conducive to the usage of hydrogen [4]. It has been reported that hydrogen exists in a chemical form and it is then released through a catalytic process, which is a promising method to solve the storage and application of hydrogen [5,6,7]. Therefore, considering the environmental impact of the hydrogen production process, the use of renewable resources, especially the liquid-phase biomass hydrogen carrier, has attracted extensive attention [8,9,10]. Among various biomass liquid hydrogen carriers, glycerol is economically attractive and environmentally friendly, because of its advantage of relatively high hydrogen content, non-toxicity, and easy storage. More importantly, the process of producing biodiesel by transesterification animal oil (or vegetable oil) and alcohols will release about 10% of crude glycerin as a byproduct [11,12,13]. With the increase of global biodiesel production capacity, crude glycerol production is obviously in excess, resulting in the continuous tendency to decrease crude glycerol price [14]. Therefore, the conversion of crude glycerol derived from bio-derived products into valuable chemicals is not only environmentally friendly but also has economic value. Glycerin and methanol are the main components of crude glycerin, among which the content of glycerol is between 40% and 85%, and the content of methanol can be as high as 25% [15]. However, there are some defects in using glycerol and methanol purified from crude glycerol and refined glycerol as biomass hydrogen carriers, such as complex processing technology, low use rate and high cost. Herein, it is of great significance to study the different ratios of glycerol and methanol mixed-alcohol feeds for the high value-added use of crude glycerol.

Noble metal catalysts (such as Pt, Ru) can effectively break the C–C and C–H bonds, lowering the reaction temperature and a considerably better stability, but the high cost limits its large-scale application [16]. Copper-based catalysts have very good low-temperature catalytic activity. They are usually used for Water−Gas Shift (WGS) reaction and CO oxidation reactions or other low-temperature reactions, but they are prone to severe sintering at high temperatures [17,18,19,20]. Ni is a non-precious metal with the ability to break C–C bonds and C–H bonds which can be used commercially as an active component for hydrogen production from shale gas and alcohol reforming [21,22,23,24,25]. However, Ni particles are prone to sintering and depositing carbon at high temperatures, leading to a decrease in catalytic performance [26]. Generally, the sintering of Ni particles can be suppressed by increasing the calcination temperature or doping the second metal, especially the rare earth elements (such as Ce, Pr) [27]. Nevertheless, an excessively high calcination temperature will cause a drastic decrease in the specific surface area of the support and the active metal. Moreover, an excessively strong metal–support interaction force will make it difficult to reduce Ni species. For supported metal catalysts, the activity and selectivity of the steam reforming reaction are largely affected by the nature of the support and its interaction with the active metal. Many works have been reported about the carriers and the corresponding reaction processes for hydrogen production reaction of alcohol reforming, including Al_2_O_3_, ZrO_2_, CeO_2_, La_2_O_3_, and TiO_2_ [21,23,28,29,30]. Among them, the non-redox carrier Al_2_O_3_ was widely used in the hydrogen production reaction of glycerol steam reforming due to its large specific surface area and good hydrothermal stability. However, it is easy to form a strong interaction force between Al_2_O_3_ and the active metal Ni, forming an inactive NiAl_2_O_4_ spinel, which is difficult to reduce to catalytically active Ni^0^ species [31]. Extensive research is mainly focused on weakening the interaction between Ni species and Al_2_O_3_ by adding rare earth elements, therefore promoting the reduction of Ni species [21,32]. Lu et al. found that the addition of rare earth element Pr can change the electronic environment around Ni species, therefore weakening the interaction between nickel and Al_2_O_3_, and strengthening the low-temperature catalytic activity of methanol steam reforming hydrogen production [21]. Compared with the Al_2_O_3_ carrier, the reducible support TiO_2_ was more widely used in the field of water–gas shift reaction, selective hydrogenation and steam reforming reactions due to the electronic effect and strong interaction between it and the active metal [33,34,35,36]. Joseph and co-workers studied the selective hydrogenation of acetophenone on a titanium-supported nickel catalyst, and found that nickel interacts with rutile electrons, resulting in an electron-rich Ni-H active species [35]. Xu et al. recent research has shown that Ti will transfer electrons to nickel, therefore producing electron-rich Ni^δ^-O_v_-Ti^3+^ (O_v_ denotes oxygen vacancy), which strengthens the activity of the water–gas shift reaction [33,34]. Compared with the non-reducible carrier Al_2_O_3_, which requires the addition of additives to control the electronic effect between it and the active metal, there is an obvious electron transfer effect between Ni and the reducible support TiO_2_. However, the influence of the electronic effect of Ni-Ti system catalyst on the catalytic performance of alcohol steam reforming hydrogen production is still unclear.

Promoted by the above demands, we tried to tune the electronic effect between Ni and the support TiO_2_ by changing the calcination temperature, and then study its influence on the steam reforming of glycerol. At the same time, considering that the crude glycerol contains a large amount of methanol, we have also studied in detail the influence of different glycerol/methanol/water ratio feeds on the reaction. To the best of our knowledge, there is no report on the electronic effect of Ni/TiO_2_ catalyst on its initial catalytic performance, as well as the influence of mixed-alcohol feeds with different volume ratios of glycerol and methanol on the reaction (GMSR).

## 2. Materials and Methods

### 2.1. Catalyst Preparation

A commercial TiO_2_ (P25, surface area = 50 m^2^/g, Degussa Inc., Hessen, Germany) without further calcination, and nickel nitrate (Ni(NO_3_)_2_·6H_2_O) were obtained from Aladdin Industrial Corporation (Shanghai, China). During the whole experimental process, deionized water was used, and the chemicals were used without further purification and pretreatment.

Typically, 1.0 g of Ni(NO_3_)_2_·6H_2_O was dispersed in 2.4 mL distilled water and then 1.8 g TiO_2_ was added into the above solution using incipient-wetness impregnation method with stirring intensely for 20 min and then ultrasonic treatment for 5 min. After impregnation, the slurry was aged at room temperature and dried at 110 °C for 12 h, respectively, and finally thermally decomposed in a static air muffle furnace under different set-temperatures with a dwell time of 5 h (5 °C/min). To explore the effect of calcination temperature on the electronic effect between Ni and reducible oxide supports TiO_2_, the catalysts were synthesized according to the above processes except for calcination temperature (500 °C, 600 °C, 700 °C, and 800 °C), which were labeled as NiO/Ti-500C, NiO/Ti-600C, NiO/Ti-700C, NiO/Ti-800C, respectively. The NiO/Ti-100C sample represents only after being aged without further calcination. The reduced catalysts were marked as Ni/Ti-500R, Ni/Ti-600R, Ni/Ti-700R, Ni/Ti-800R, respectively.

### 2.2. GSR Catalyst Testing

First, the GSR was used as a probe reaction to study the catalytic performance of the above catalysts, and then GMSR was studied on the best catalyst. Those reactions were performed in a fixed-bed quartz reactor (Chengtai Quartz Inc., Jiangsu, Chnia) with an inner diameter of 8 mm under normal pressure. The reactor was equipped with 200 mg of catalyst (40–60 mesh) and mixed with 100 mg of quartz particles. Prior to each test, the samples were in situ reduced at 500 °C (NiO/Ti-500C) or 600 °C (NiO/Ti-600C), 700 °C (NiO/Ti-700C), 800 °C (NiO/Ti-800C) under a flow of gaseous mixture of H_2_/N_2_ (50 vol%) with the flow rate of 60 mL/min, then kept for 1 h. Then a glycerol (30 wt. %)/water solution was fed (0.03 mL/min) by a HPLC (Japan Spectroscopy Inc., PU980, Tokyo, Japan) pump and then was vaporized at 220 °C, and the inert N_2_ (N_2_/feed = 4/1) was also introduced into the vaporizer(Chengtai Quartz Inc., Jiangsu, China) at the same time. The corresponding weight hourly space velocity (WHSV) was fixed at 9 h^−1^. The products were first condensed through a cooler, and the incondensable gas species were analyzed online by two gas chromatographs with two thermal conductivity detectors (Fuli Analytical Instrument Inc., Hangzhou, China). For activity testing, reaction temperature ranged from 500 °C to 700 °C with an interval of 50 °C, and the corresponding activity data were averaged from three recorded data at each test temperature range. The stability tests of all samples were performed at 600 °C for 20 h with the same conditions. Considering that in the liquid product obtained by condensation, there are less C_2_ and C_2+_ compounds. Therefore, the catalytic performance of the catalyst is presented in terms of the gaseous conversion rate of glycerol and glycerol–methanol mixed alcohol for GSR and GMSR, respectively, the molar yield of H_2_ and the selectivity of carbon-containing gas products.

The conversion was calculated by

(1) $$X_{\mathrm{glycerol}}=\frac{{\mathrm{Carbon}}_{g}}{3\times {\mathrm{glycerol}}_{\mathrm{in}}}\times 100\%$$

(2) $$X_{\mathrm{mixed}\;\mathrm{alcohol}}=\frac{\begin{array}{ccc} \begin{array}{ccc} \begin{array}{cc} \mathrm{moles} & \mathrm{of} \end{array} & \mathrm{carbon} & \mathrm{in} \end{array} & \mathrm{gas} & \mathrm{production} \end{array}}{3{\mathrm{glycerol}}_{\mathrm{in}}+{\mathrm{methanol}}_{\mathrm{in}}}\times 100\%$$

The glycerol_in_, methanol_in_ and carbon_g_ mean the moles of glycerol fed in, methanol fed in and carbon detected in gaseous products, respectively.

The H_2_ mole yield was calculated by

(3) $$Y_{H_{2}}=\frac{\begin{array}{ccc} \begin{array}{cc} \mathrm{moles} & \mathrm{of} \end{array} & H_{2} & \mathrm{productions} \end{array}}{{\mathrm{glycerol}}_{\mathrm{in}}}$$

The selectivity of carbonaceous gaseous products (CO, CO_2_, CH_4_) were calculated by

(4) $$S_{i}=\frac{\begin{array}{cccc} & \mathrm{moles} & \mathrm{of} & i \end{array}}{\begin{array}{ccc} \begin{array}{ccc} \begin{array}{cc} \mathrm{moles} & \mathrm{of} \end{array} & \mathrm{carbon} & \mathrm{in} \end{array} & \mathrm{gas} & \mathrm{production} \end{array}}\times 100\%$$

S_i_ represents the selectivity of CO, CO_2_, CH_4_ in the product, respectively.

### 2.3. Catalyst Characterization

Structural properties of the catalysts were carried out by N_2_ adsorption–desorption (Quantachrome NOVA 4200e, Kantar Technology Inc., New York, NY, USA). All samples were pretreated at 300 °C for 3 h. Specific surface area (BET) was obtained from the data of adsorption branch at −196 °C using the BET method, and pore size distribution was calculated from Barrett–Joyner–Halenda (BJH) method. X-ray powder diffraction (XRD) (Japan Rigaku Inc., Tokyo, Japan) patterns were measured by a Rigaku D/max-1200 diffractometer equipped with Cu K*α* radiation (*λ* = 0.15406 nm), operated at 30 mA and 40 kV. The crystalline size of Ni and NiO were calculated with Scherer equation using the Rietveld refinement of Jade 6.0 software. The UV-vis diffuse reflection spectrum was recorded by the TU-1901 UV-vis spectrophotometer(Puxi Technology Inc., Beijing, China) produced by the Persee General Instrument Co., Ltd. and equipped with an integrating sphere. The X-ray photoelectron spectra (XPS) was carried out on an ESCALAB 250 Xi spectrometer (Thermo Scientific Inc., Waltham, MA, USA) equipped with an Al K*_α_* (h*ν* = 1486.6 eV) X-ray source. The charging effect was corrected by adjusting the binding energy of the C 1s peak from carbon pollution to 284.8 eV. Raman spectra were carried out on a micro-Raman system (Dilor Technology Inc., Paris, France) equipped with a 532 nm laser as the excitation source. A thermogravimetric analyzer (TGA 4000, PerKinElmer Inc., TGA 4000, Waltham, MA, USA) was used to analyze coke deposition on the spent catalysts. NH_3_-Temperature programmed desorption (NH_3_-TPD) experiment was performed on a TCD detect (Fuli Analytical Instrument Inc., Hangzhou, China) to explore the nature of acidity on catalysts. Before recording the TPD profiles, 100 mg of fresh catalyst was pretreated under 450 °C for 1 h and cooled down to 100 °C in the flow of ultra-pure He, then the gas was switched to NH_3_ flow at 30 mL/min for 1 h. After that, the gas was switched to ultra-pure He flow at 30 mL/min to purge the material for 2 h, finally, the pretreated sample was heated to 800 °C under the ultra-pure He flow at a heating rate of 10 °C/min. H_2_-Temperature programmed reduction (H_2_-TPR) (Homemade, Kunming, China) experiment was performed to investigate redox properties of catalysts. In short, in a typical test, a sample (50 mg) is first pretreated with a gas mixture (5 vol.% O_2_ in Ar) at 400 °C for 1 h to remove moisture and impurities. After cooling down to 100 °C, the gas was switched to the mixed gas flow with 10% H_2_/Ar (30 mL/min). The pretreated catalyst was heated to 800 °C at a rate of 10 °C/min and the TCD signal was recorded continuously. The H_2_ chemisorption experiment was used to measure the ratios of H_2_/Ni for the four catalysts. Prior to hydrogen pulse chemisorption, catalysts were pretreated at the reduction temperature under 10% H_2_/Ar (30mL/min), and cooled down to 40 °C in the flow of ultra-pure He. The chemisorption was implemented by pulsing of a mixture of 10% H_2_/Ar (30 mL/min).

## 3. Results and Discussion

### 3.1. Physicochemical Properties of the Samples

**Figure 1:**
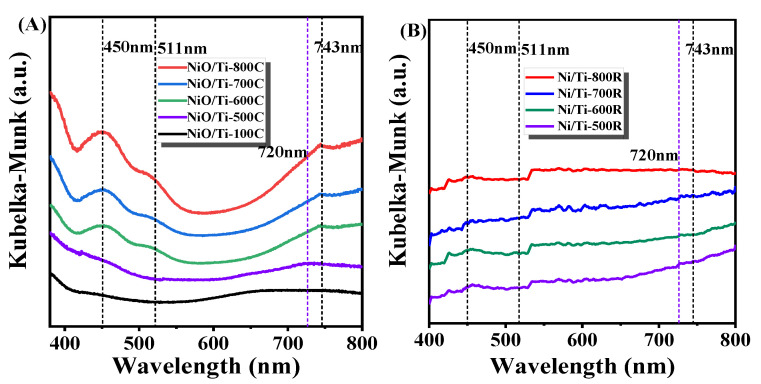
UV−vis diffuse reflection spectrum of the samples annealing (**A**) and reduce (**B**).

UV−vis diffuse reflection spectrum (UV−vis DRS) was performed to investigate the nature of the Ni species within the material with the change of calcination temperature. The DR spectra of the samples with different calcination temperatures are shown in Figure 1A. The peaks of the NiO/Ti-100C sample at 650 nm and 750 nm in the visible region can be attributed to the peak of Ni^2+^ ions adsorbed on the surface of TiO_2_, which disappears in the ultraviolet region. Compared with NiO/Ti-100C sample, the peak of the NiO/Ti-500C sample at 720 nm can be attributed the species of NiO [27]. In contrast to the above samples, the NiO/Ti-600C sample shows new peaks at wavelengths 450, 511, and 743 nm, which are attributed to the NiTiO_3_ phase [37,38,39]. The spectra at 450 and 511 nm are due to the splitting of the crystal field of the 3d^8^ band of Ni^2+^ ions into two sub-bands, called Ni^2+^→Ti^4+^ charge transfer (CT) bands. It also shows absorbance at 743 nm as indicated by its yellow color [38]. The reduction catalysts were also characterized by the UV−vis diffuse reflection spectrum shown in Figure 1B. The peaks of NiTiO_3_ and NiO disappear, indicating that NiO and NiTiO_3_ are completely reduced to Ni^0^. The above observations clearly indicate that the interaction between Ni species and the support TiO_2_ gradually increases with the increase of calcination temperature, resulting in the evolution of Ni species from Ni^2+^ species adsorbed on the surface of the carrier to NiO species, and then to NiTiO_3_ spinel species.

**Figure 2:**
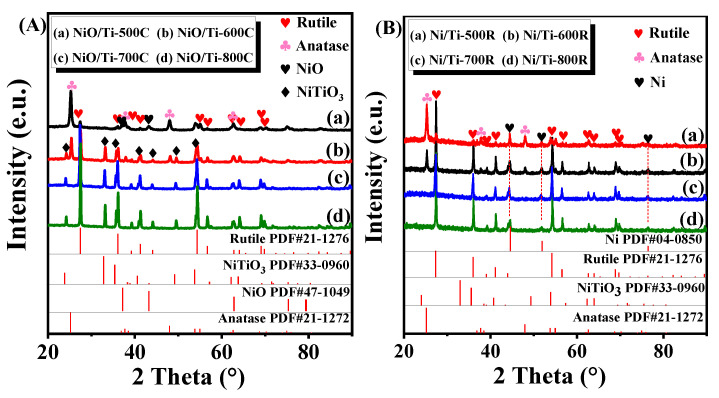
XRD patterns of the (**A**) calcined and (**B**) reduced catalysts.

We turned to the XRD to further determine the bulk composition and the average crystallite size of the above catalysts. The XRD patterns for the support and samples before and after reduction are shown in Figure 2A,B, and the calculated average crystallite size is listed in Table 1. The anatase (JCPDS#21-1272) and rutile (JCPDS#21-1276) structure existed in all samples. However, the face-centered cubic of structure NiO (JCPDS#47-1049) only appears in NiO/Ti-500C and the NiTiO_3_ species (JCPDS#33-0960) is apparent in NiO/Ti-600C, NiO/Ti-700C, NiO/Ti-800C. The characterization results are consistent with those of a UV-vis DR spectrum. It has been reported that higher calcination temperature could enhance the immersion of Ni^2+^ into TiO_2_ crystal lattices to form NiTiO_3_ crystals, which, after activation, could allow the existence of Ni crystallites highly dispersed in the support, as also previously reported [28,40]. As can be seen from Table 1, with the increase of calcination temperature, anatase gradually evolves into rutile, and the proportion of rutile in the reduction process will further increase, but the transformation speed is obviously lower than that of calcination in muffle furnace. The particle size of nickel-containing compounds for the above catalysts increases gradually and is approximated to be 15.0, 33.7, 45.5, and 76.1 nm for NiO/Ti-500C, NiO/Ti-600C, NiO/Ti-700C, NiO/Ti-800C, respectively. After catalyst reduction, the particle size of Ni^0^ increases gradually and is approximated to be 18.80 ± 1, 19.77 ± 0.5, 20.83 ± 1, and 23.25 ± 2 nm for Ni/Ti-500R, Ni/Ti-600R, Ni/Ti-700R, Ni/Ti-800R, respectively. The XRD spectrum of Ni/TiO_2_-500R catalyst after high temperature reaction is shown in Figure S1, the particle size of Ni^0^ increases slightly. However, in the case of TiO_2_ crystallite size, a monotonic increase in TiO_2_ crystallite size can be observed with increasing calcination temperature from 500 to 800 °C. After catalyst reduction, the particle size of TiO_2_ is almost the same as that after calcination, indicating that the carrier TiO_2_ is seriously sintered in the process of high-temperature calcination. At the same time, it can be seen from Figure 2B that NiO and NiTiO_3_ were not detected in the reduced catalyst, indicating that these two substances can be completely reduced to Ni^0^ specie, and the results were consistent with the UV-vis DRS results.

**Figure 3:**
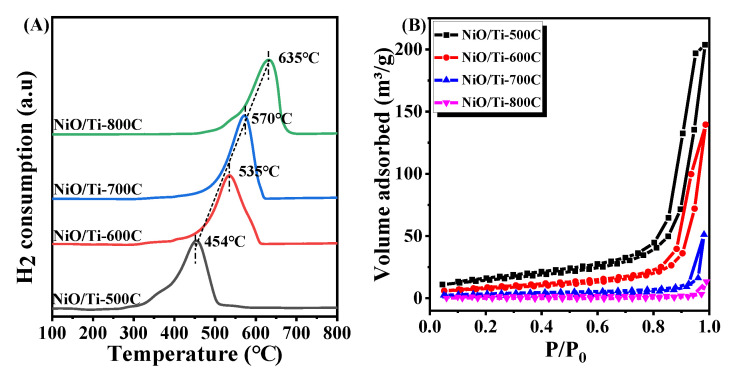
H_2_-TPR (**A**) and N_2_ adsorption–desorption isotherms (**B**) profiles of the catalysts.

To further investigate the reducibility of Ni species over NiO/Ti-500C, NiO/Ti-600C, NiO/Ti-700C, NiO/Ti-800C, H_2_-temperature programmed reduction (H_2_-TPR) was performed (Figure 3A). The main peak of NiO/Ti-500C centered at 455 °C is attributed to the reduction of highly dispersed NiO phase [34]. Compared with NiO/Ti-500 sample, hydrogen consumption peak of the NiO/Ti-600 sample shifts to higher reduction temperature and the main peak is approximated to be 535 °C and the peak is attributed to the reduction of granular NiTiO_3_ phase in medium intensity interaction with TiO_2_ [41,42]. As the calcination temperature increases, hydrogen consumption peaks of the NiO/Ti-700C and the NiO/Ti-800C samples shift to higher reduction temperature and the main peaks are approximated to be 570, 635 °C. The peaks are attributed to the reduction of interface NiTiO_3_ phase in strong interaction with support TiO_2_ or metal–support solid solution [43,44], suggesting the interface NiTiO_3_ or metal–support solid solution phase is more difficulty to reduce than NiO with weaker interaction with support. To better understand the number of active Ni species on the catalyst, H_2_ chemisorption characterization was carried out, the data are listed in Table 2. It can be seen from Table 2 that for Ni/Ti-500R, Ni/Ti-600R, Ni/Ti-700R and Ni/Ti-800R, the ratios of the four catalysts are calculated to be 74.4, 110.2, 35.6 and 25.8 μmol_H2_/g_cat_, respectively. The results indicate that Ni/Ti-600R catalyst has the best initial activity. N_2_ adsorption/desorption isotherms curves of the NiO/Ti-500C, NiO/Ti-600C, NiO/Ti-700C, NiO/Ti-800C catalysts were displayed as Figure 3B. All samples exhibit the physisorption isotherms of Type II and hysteresis loops of Type H1 [45], indicating a non-porous or macroporous structure of the catalyst particles. The data of Brunauer–Emmett–Teller (BET) surface area and respective pore volume for calcined and reduced samples are shown in Table 1. The BET surface area of NiO/Ti-500C is 64.56 ± 1 m^2^/g, which is greater than the 0.98 ± 0.3 m^2^/g of NiO/Ti-800C sample. Compared with the calcined catalysts, the specific surface area of Ni/Ti-500R sample decreases to 41.38 ± 1 m^2^/g and Ni/Ti-600R to 15.21 ± 0.5 m^2^/g. The specific surface areas of Ni/Ti-700R and Ni/Ti-800R increase slightly, and the pore volume of all samples decreases slightly. N_2_ adsorption–desorption isotherms profile of Ni/TiO_2_-500R catalyst after high temperature reaction is shown in Figure S2, and the specific surface area has decreased significantly. Combined with the XRD characterization, it can be seen that increasing the annealing temperature of the sample will cause the sintering of the supported TiO_2_ particles, which will further reduce the specific surface area and pore volume of the catalyst.

**Figure 4:**
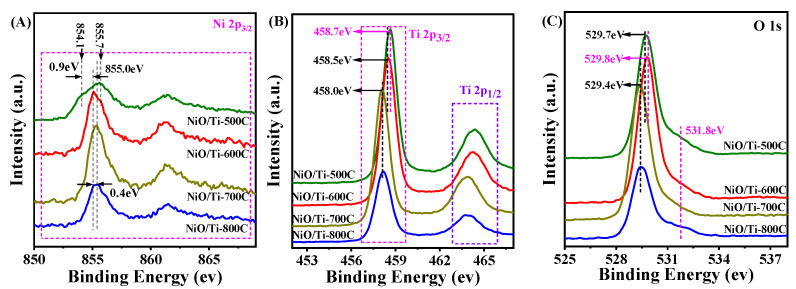
XPS spectra of Ni 2p (**A**), Ti 2p (**B**) and O 1s (**C**) for the above samples.

XPS spectra was used to prove the possible existence of electrons interaction between Ti oxide and separated NiO species. The corresponding Ni 2p, Ti 2p, and O 1s XPS spectra of NiO/Ti-500C, NiO/Ti-600C, NiO/Ti-700C, NiO/Ti-800C catalysts, respectively, are given in Figure 4. For the NiO/Ti-500C catalyst, 855.7 eV can be attributed to Ni^3+^, it may be that NiO is oxidized to Ni_2_O_3_ during the calcination process, or the interaction between the Ni precursor and the support TiO_2_ is weak at a lower calcination temperature, and it decomposes into NiO and Ni_2_O_3_ [46,47]. The electron cloud density around Ni^3+^ is low, so the binding energy shifts to higher binding energy relative to Ni^2+^ in NiO. The binding energy of 854.1 eV is attributed to the weaker Ni^2+^ peak that interacts with the carrier TiO_2_ [46]. As the calcination temperature of the catalysts increases, the interaction between NiO and the support TiO_2_ becomes stronger, and the electronic environment around the Ni species changes significantly. Compared with Ni^2+^ (854.1 eV) in NiO/Ti-500C catalyst, the binding energy of Ni^2+^ in Ni 2p XPS spectrum for NiO/Ti-600C (855.0 eV), NiO/Ti-700C and NiO/Ti-800C (ca. 855.4 eV) catalyst shifts to high binding energy but are 0.3 eV lower than Ni^3+^. As for the Ti 2p and O 1s XPS spectra, compared with the NiO/Ti-500C catalyst, with the increase of the catalyst calcination temperature, except for the binding energy of O 1s spectrum for NiO/Ti-600C catalyst slightly shifts to higher binding energy, the binding energies of Ti 2p and O 1s XPS spectra for all catalysts shift to low binding energies. This indicates that when the calcination temperature is increased, there is a stronger electronic activity phenomenon between the carrier TiO_2_ and the active metal Ni species. These changes may also be caused by anatase—rutile phase transition in the carrier [48].

Combined with XRD, UV-vis DRS, H_2_-TPR and BET characterization results, increasing the calcination temperature, the interaction force and the electronic effect between the active metal Ni and the support gradually increase, and the interaction force between NiTiO_3_ and the support TiO_2_ is also enhanced. For NiO/Ti-600C catalyst, the active metal Ni and the support TiO_2_ form a medium-strength interaction force and electronic effect, which leads to the existence of the Ni species in the catalyst in the form of granular NiTiO_3_, which is easily reduced to small particles of Ni at relatively lower temperatures. However, under high-temperature calcination, a very strong interaction force and electronic effect are formed between interface NiTiO_3_ and the support TiO_2_, or in the form of a Ni-Ti solid solution, which caused the Ni^2+^ in NiTiO_3_ to be reduced at a higher temperature. The higher calcination and reduction temperature will cause severe sintering of the active metal and the carrier, therefore affecting the performance of the catalyst.

**Figure 5:**
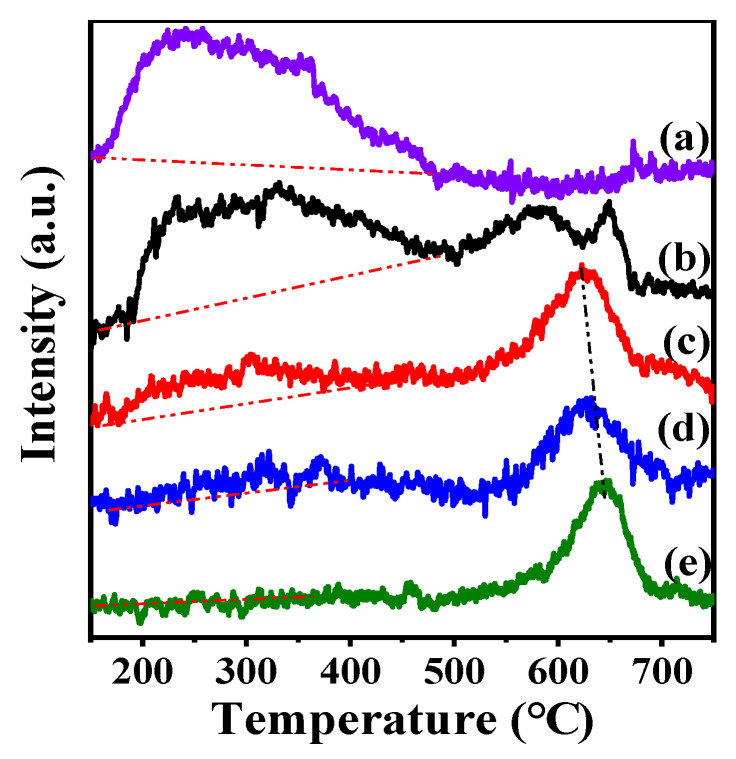
NH_3_-TPD profiles of (**a**) TiO_2_, (**b**) NiO/Ti-500C, (**c**) NiO/Ti-600C, (**d**) NiO/Ti-700C, (**e**) NiO/Ti-800C.

It is well known that the acidity of the catalyst has an important influence on the catalytic performance and anti-coking performance during the reforming reaction [49]. Weak acid facilitates the adsorption of reactants, while strong acid easily led to carbon on the catalyst surface. NH_3_-Temperature programmed desorption profile was used to study the acid strength characteristics of the Ni-supported TiO_2_ catalyst as a function of the calcination temperature. The results are shown in Figure 5 and Table 2. It can be stated that all catalysts are dominated by two desorption regions at lower than 550 °C and higher than 550 °C associated with weak/medium and strong acid sites, respectively [50]. For the support TiO_2_, there is a bulky and wide desorption peak (T_max_ = 220 °C) caused by Ti–OH of the support surface, indicating that the weak acid and medium strong acid coexist in the support TiO_2_ and the weak acid is the main one. For the NiO/Ti-500C, the intensity of desorption peak at 350 °C increases gradually, and new desorption peaks appear at ca. 550 °C and ca. 650 °C, indicating that the acidity of the catalyst becomes stronger when the catalyst is calcined at 500 °C. For the NiO/Ti-600C, NiO/Ti-700C, NiO/Ti-800C, when the calcination temperature is further increased, the intensity of desorption peak below 550 °C gradually decreases or even disappears, while the desorption peak above 550 °C shifts to a higher temperature, indicating that the NiO/TiO_2_ catalyst transforms weak/medium acid into strong acid at higher temperature. As we all know, the strong acidity of the catalyst may cause carbon deposition on the surface of the catalyst, which will lead to a decrease in its catalytic activity [51]. The effect of the surface acidity on the catalytic properties will be discussed below.

### 3.2. Catalytic Performance Tests

#### 3.2.1. Catalytic Performance Tests of GSR

**Figure 6:**
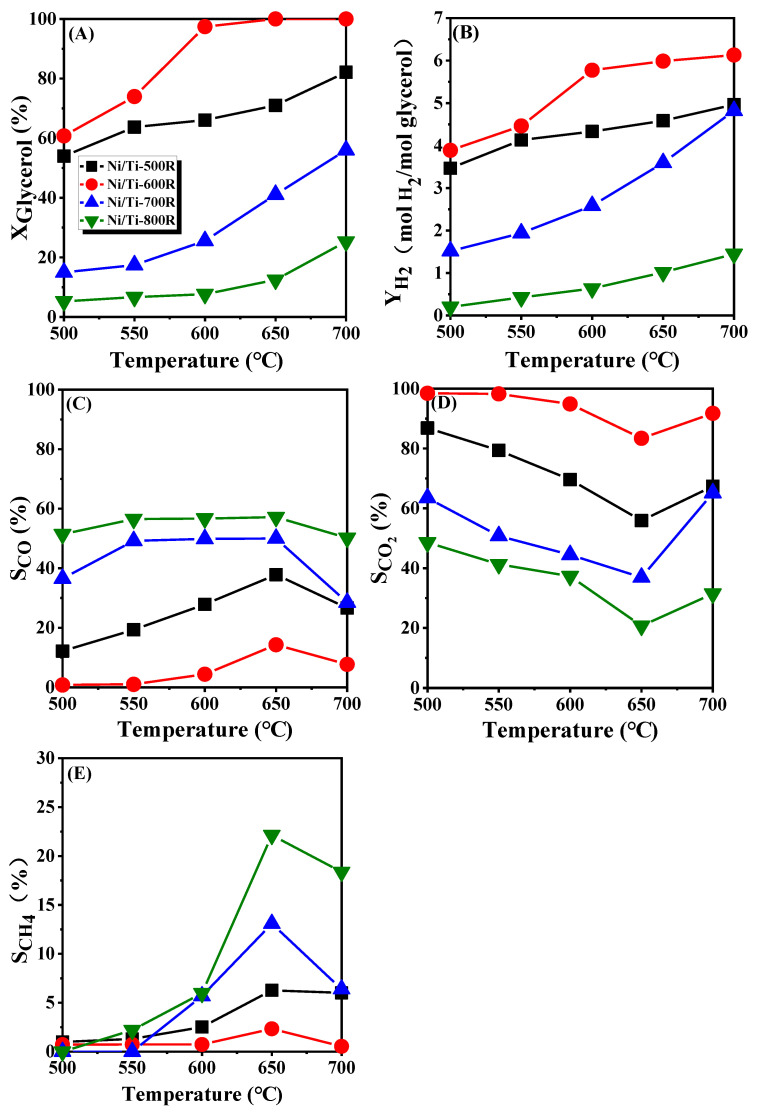
GSR activity of the catalysts (**A**) glycerol conversion, (**B**) H_2_ yield, (**C**) CO selectivity, (**D**) CO_2_ selectivity, (**E**) CH_4_ selectivity. Reaction conditions: 30 wt.% glycerol/water solution, WHSV = 9 h^−1^, 1 atm.

The effects of reaction temperature on the activity of Ni/Ti-500R, Ni/Ti-600R, Ni/Ti-700R and Ni/Ti-800R in GSR were investigated, and the corresponding results are shown in Figure 6. When the reaction temperature is 500 °C and 550 °C, the glycerol conversion rate and H_2_ yield of all catalysts are low, indicating that the Ni particles have a weak ability to break chemical bonds at low temperatures [52]. Moreover, glycerol conversion and H_2_ yield of Ni/Ti-500R and Ni/Ti-600R are higher than those of Ni/Ti-700R and Ni/Ti-800R catalysts under the same reaction temperature. At 600 °C, the conversion rate of glycerol reaches 98.5% for Ni/Ti-600R catalyst, but the conversion rate of other catalysts is much lower than that of Ni/Ti-600R catalyst. When the reaction temperature reached 650 °C and 700 °C, the glycerol conversion rate of Ni/Ti-600R catalyst is almost completely achieved, but the glycerol conversion rate of other catalysts was still far lower than Ni/Ti-600R. For all the catalysts, the conversion rate of glycerol and the yield of hydrogen increased gradually with the increase of the reaction temperature. For Ni/Ti-600R catalyst, the conversion rate of glycerol and the yield of hydrogen increased rapidly between 550 °C and 600 °C, and then the increase of temperature did not influence much. With the increase of reaction temperature, the selectivity of CO and methane increases first and then decreases, reaching the maximum at 650 °C. However, the selectivity of CO_2_ decreased first and then increased, and the selectivity is the lowest at 650 °C. The above experimental phenomena accord with the reaction thermodynamics. The lower reforming temperature limits the catalytic performance of the catalyst. The feed mainly forms liquid carbon compounds or coke deposits, resulting in a decrease in gaseous products and a decrease in H_2_ yield [53].

**Figure 7:**
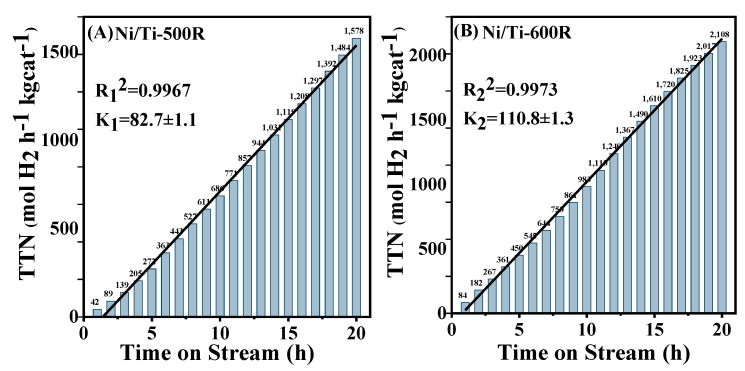
Stability test of (**A**) Ni/Ti-500R and (**B**) Ni/Ti-600R catalysts for hydrogen production in 20 h.

As the temperature increased, the more gas was generated, and the reforming process is more favorable. In addition, as the temperature goes up, the WGS reaction and methanation reaction tends to proceed in the opposite direction, since these two reactions are exothermic reactions [54]. Hence, when the temperature is higher than 650 °C, CO selectivity increases and CH_4_ selectivity decreases. When the reaction temperature is lower than 650 °C, although the high temperature will inhibit the methanation reaction, the glycerol conversion rate will increase, and more CO will be produced during the reaction. In the process of increasing the temperature from 500 °C to 650 °C, from the perspective of reaction thermodynamics, increasing the reaction temperature will inhibit the methanation reaction of CO (CO + 3H_2_ = H_2_O + CH_4_ ΔH_0_ = −206.11 kJ/mol). However, as the reaction temperature increases, the glycerol conversion rate increases, and more CO will be produced, resulting in an increase in the selectivity of methane in the product. When the temperature is higher than 650 °C, CO selectivity and CH_4_ selectivity in the product decreases. Comparing the catalytic performance of the above catalysts, increasing the calcination temperature of the catalyst during the preparation process results in the catalytic activity order of Ni/Ti-600R > Ni/Ti-500R > Ni/Ti-700R > Ni/Ti-800R catalysts and the result is consistent with the H_2_/Ni ratio characterized by H_2_ chemisorption. Compared with Ni/Ti-600R catalyst, the Ni/Ti-700R and Ni/Ti-800R catalysts have lower glycerol conversion rate and hydrogen production rate, which may be due to their lower specific surface areas and stronger acidity. At the same time, the strong acidity of the catalyst promotes the cracking reaction of glycerol, resulting in a significantly higher CO selectivity in the product than Ni/Ti-500R and Ni/Ti-600R catalysts. Therefore, Ni/Ti-600R and Ni/Ti-500R are used to investigate the stability of the catalyst at the reaction temperature of 600 °C, and the hydrogen production capacity is shown as the index listed in Figure 7. In a 20-h test, the Ni/Ti-600R and Ni/Ti-500R catalyst achieved a total turnover number of 2108 mole and 1578 mole hydrogen per kilogram catalyst. In terms of linear correlation and slope for Ni/Ti-600R and Ni/Ti-500R, respectively, R_2_^2^ (0.9973) is larger than R_1_^2^ (0.9967), and K_2_ (110.8 ± 1.3) is larger than K_1_ (82.7 ± 1.1), indicating that the stability of hydrogen production capacity of Ni/Ti-600R catalyst is higher than that of Ni/Ti-500R catalyst.

#### 3.2.2. Catalytic Performance Tests of GMSR

**Figure 8:**
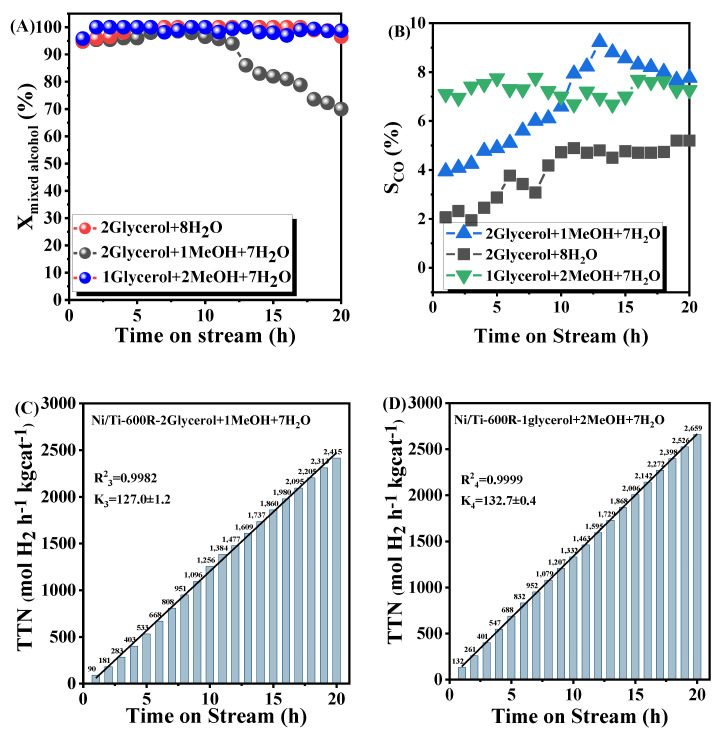
The conversion rate (**A**), CO selectivity (**B**) and hydrogen production stability (**C**), (**D**) of mixed alcohol feeds on Ni/Ti-600R catalyst in 20 h.

Considering that the byproduct crude glycerin of biodiesel itself contains about 25% methanol by mass, the adaptability of the Ni/Ti-600R catalyst to reactant systems with different methanol/glycerol/water volume ratios was further investigated. The results are shown in Figure 8. It can be seen from Figure 8, compared with the feed glycerol/methanol/water ratio of 2/0/8 and 2/1/7, adding a certain amount of additional methanol into the original glycerol system will lead to a decrease in the conversion rate of mixed alcohol and an increase in CO selectivity. However, the hydrogen production capacity will not decrease, and the hydrogen production capacity within 20 h is 1.15 times that of the single glycerol system. The decrease of mixed-alcohol conversion rate may be caused by the increase of Ni/Ti-600R catalyst substrate due to the addition of a certain amount of methanol. There is competitive adsorption between small molecule methanol and glycerol. As the reaction time increases, the catalytic ability of the catalyst decreases, and glycerol may dehydrate to produce aldehydes. This process is likely to cause the formation of graphitized carbon on the surface of the catalyst, which is difficult to remove, resulting the activity of the catalyst gradually decreases [55]. Compared with the feedstock glycerol/methanol/water ratio of 2/0/8 and 1/2/7, the mixed alcohols of the two feedstocks are almost completely converted within 20 h. However, hydrogen production and CO concentration in gas products increased significantly when methanol content is higher. Therefore, in the mixed-alcohol reaction system, increasing the ratio of methanol to glycerol can increase the concentration and stability of hydrogen in the product, but compared with the single glycerol feed, the selectivity of CO in the gas product will also increase.

## 4. Mechanism Analysis of Catalyst Deactivation

The deactivation of catalysts in the GSR (GMSR) reaction is mainly caused by carbon deposition and sintering of active species Ni, among which many researchers reported that the most important reason is the carbon deposition. Therefore, thermogravimetry and Raman were used to characterize and analyze the amount and type of carbon on the surface area of Ni/Ti-600R catalyst after 20 h reaction in different mixed-alcohol systems.

**Figure 9:**
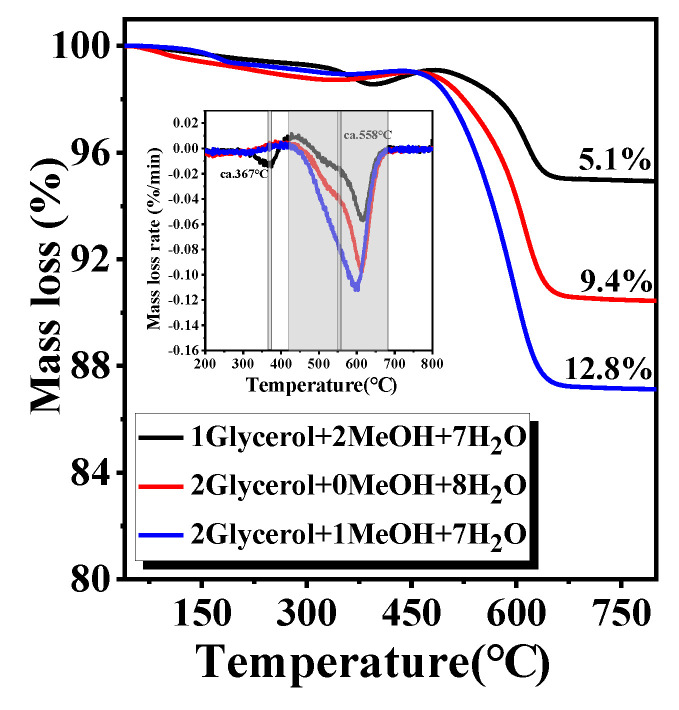
TGA and DTG (inset) profile of the Ni/Ti-600R catalyst for different mixed-alcohol feed after 20 h stability test.

Thermogravimetric analyzer (TGA) was used to characterize the coking characteristics and coking amount of spent catalyst and the results are shown in Figure 9. The initial losses in the 150–300 °C region are attributed to the removal of easily oxidized carbonaceous species, such as adsorbed reactants or liquid products [56]. Compared with the feedstock glycerol/methanol/water ratio of 2/0/8 and 2/1/7, the glycerol/ methanol/ water ratio of 1/2/7 feedstock with higher methanol ratio appears a loss peak at 367 °C, which can be attributed the gasification of weakly stable amorphous (C*_α_*) [57]. The major loss peak located at the range of 300–660 °C, and the corresponding differential peaks are shown in the inset profile in Figure 9. The three feeds have similar differential peak centered at ca. 595 °C, which is probably due to the gasification of the bulky carbonaceous species or the graphitized carbon on spent catalysts [56]. The feedstock of glycerol/methanol/ water ratio of 2/0/8 and 2/1/7 appear an additional shoulder peak at 558 °C which can be attributed to the gasification of filamentous coke (C*_β_*) with different graphitization degree [57]. As listed in Table 3, the mass losses assigned to coke gasification after 20 h reaction on the feedstock glycerol/methanol/ water ratio of 1/2/7, 2/0/7 and 2/1/7 are 5.1%, 9.4% and 12.8% respectively. The above phenomena indicate that the glycerol/ methanol/ water ratio of 1/2/7 feedstock with higher methanol ratio forms carbon types that are easier to remove during the reaction process, resulting in lower carbon accumulation.

**Figure 10:**
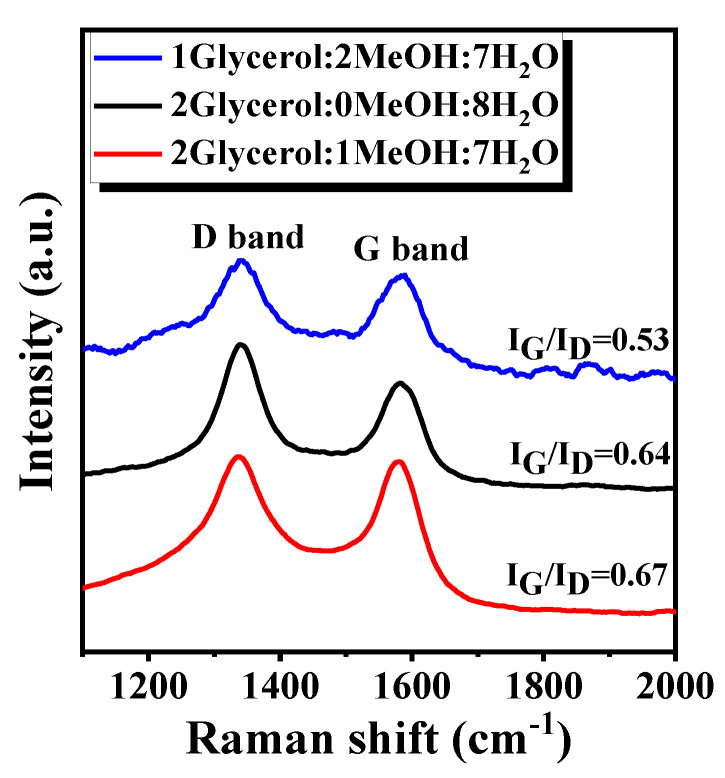
Raman spectrum of the Ni/Ti-600R catalyst for different mixed-alcohol feed after 20 h stability test.

The Raman spectrum was applied to further analyze the type of coke deposition on spent catalysts used in different mixed-alcohol feeding systems, and the Raman spectrum in the range 1100–2000 cm^−1^ is presented in Figure 10 and Table 3. The spent catalysts used in different mixed-alcohol feeding systems display peaks around 1350 cm^−1^ and 1590 cm^−1^ in Raman spectra, which are categorized as D-band and G-band of carbon-based materials, respectively. D-band, which is usually caused by the vibration of carbon atoms with dangling bonds in disordered carbon-like substances, and G-band is attributed to the well-ordered, condensed or graphitic aromatic carbon species as the sp^2^ bonded carbon atoms in a two-dimensional hexagonal lattice [27]. In addition, the ratio of band intensities (I_G_/I_D_) has been used to characterize the graphitic degree of coke on catalysts [58]. The ratios of I_G_/I_D_ of Ni/Ti-600R after 20 h reaction on the feedstock glycerol/methanol/water ratios of 1/2/7, 2/0/7 and 2/1/7 are 0.53, 0.64 and 0.67, respectively. Raman results are consistent with TG characterization, which indicate that the mixed-alcohol feed with a glycerol/methanol/water ratio of 1/2/7 deposits more unstable amorphous (C*_α_*) and filamentous coke (C*_β_*) on the catalyst. These carbons are easy to gasify and decompose in the reaction process at high temperature, so TG characterization shows that the amount of carbon deposition is minimal under this feed condition.

## 5. Conclusions

In our work, we found that the calcination temperature of the Ni/TiO_2_ catalyst during the preparation process has a great influence on its physical and chemical properties and the catalytic performance of the corresponding alcohol reforming hydrogen production reaction. During the preparation process of the Ni/TiO_2_ catalyst, compared to calcination at 500 °C to form NiO, calcination at 600 °C forms a granular NiTiO_3_ species with medium intensity interaction and electronic activity between the metal and support. Due to the moderate metal–support interaction and electronic activity for NiO/Ti-600C catalyst, the granular NiTiO_3_ spinel can be reduced to a smaller Ni^0^ at a lower temperature, showing the best glycerol conversion rate and the highest hydrogen yield. However, when the catalyst is calcined at 700 and 800 °C, the Ni species exists in the form of interface NiTiO_3_ spinel or Ni-Ti solid solution with strong metal–support interaction force and electronic effect, which requires a higher temperature to reduce to Ni^0^ species. The higher calcination and reduction temperature will cause severe sintering of the active metal and the carrier, therefore affecting the performance of the catalyst. At the same time, as the calcination temperature increases, the acidity of the catalyst gradually changes from weak acid to strong acid.

We have also carried out research on hydrogen production by reforming mixed alcohols with different ratios of glycerol/methanol/water as feedstock on Ni/Ti-600R catalyst. It is found that when the concentration of glycerol is the same, adding a certain amount of methanol will increase the amount of carbon deposit on the catalyst, and the conversion rate of mixed alcohol will decrease, but the hydrogen production will increase. When the methanol concentration in the mixed-alcohol feed is the highest, the carbon deposit is the least, and the hydrogen production will further increase. Therefore, for crude glycerin, a byproduct of biodiesel, a certain amount of methanol will help increase hydrogen production. When the volume ratio of glycerol to methanol is less than 50%, it will not only help reduce carbon deposits, but also increase hydrogen production.

## Figures and Tables

**Table 1:** Physicochemical parameters for the samples.

Sample	The Proportion of Rutile (%)	Crystallite Size of Rutile [110] ^a^ (nm)	Crystallite Size of NiO [111] ^a^ (nm)	Crystallite Size of NiTiO3 [104] ^a^ (nm)	Crystallite Size of Ni [200] ^a^ (nm)	BET Surface Area (m^2^/g) ^b^	Pore Volume (cm^3^/g)	Pore Diameter (nm)
NiO/Ti-500C	11.5	27.3	14.97	_	_	64.56 ± 1	0.32	13.38
NiO/Ti-600C	54.0	36.2	_ ^c^	33.70	_	33.00 ± 0.5	0.22	15.31
NiO/Ti-700C	100.0	50.0	_	46.50	_	7.46 ± 0.5	0.08	3.25
NiO/Ti-800C	100.0	74.9	_	67.10	_	0.98 ± 0.3	0.02	3.92
Ni/Ti-500R	13.7	33.0	_	_	18.80 ± 1	41.38 ± 1	0.24	11.17
Ni/Ti-600R	71.2	44.3	_	_	19.77 ± 0.5	15.21 ± 0.5	0.11	14.56
Ni/Ti-700R	100.0	49.1	_	_	20.83 ± 1	_	_	_
Ni/Ti-800R	100.0	75.0	_	_	23.25 ± 2	1.18 ± 0.2	0.01	4.28

^a^ Crystallite size was determined by XRD with the Scherrer equation; ^b^ Determined by N_2_ adsorption–desorption, calcined catalyst/reduced catalyst; ^c^ no data.

**Table 2:** NH_3_ desorbed and H_2_ uptake of samples.

Samples	NH_3_ Desorbed (**μ**mol/g_cat_)			H_2_ Uptake (**μmol_H2_/g_cat_**)
	T < 550 °C	T > 550 °C	Total	
TiO_2_	313.2	7.1	320.3	-
Ni/Ti-500C	265.2	80.1	345.3	74.4
Ni/Ti-600C	114.7	149.3	264.0	110.2
Ni/Ti-700C	47.8	127.6	175.4	35.6
Ni/Ti-800C	5.4	113.7	119.1	25.8

**Table 3:** Quantitative calculation of coke deposition on spent catalyst in 20 h.

Feed Glycerol/MeOH/H_2_O	Weight Loss (%)	Coke/Glycerol (mmol/mol)	Coke Formation Rate mol/gcat/s	Carbon Balance (%)	I_G_/I_D_
1/2/7	5.1	0.25	5.9 × 10^−8^	99.1	0.53
2/0/8	9.4	0.37	1.1 × 10^−7^	99.1	0.64
2/1/7	12.8	0.44	1.5 × 10^−7^	89.2	0.67

## Data Availability

All data used to support the findings of this study are included within the article.

## Supplementary Materials

The following are available online at https://www.mdpi.com/article/10.3390/nano11113149/s1, Figure S1. N_2_ adsorption–desorption isotherms profile of the Ni/Ti-500R catalysts after 20 h stability test. Figure S2. XRD pattern of the Ni/Ti-500R catalysts after 20 h stability test.
