# Supplementary material for: Study of the Metal–Support Interaction and Electronic Effect Induced by Calcination Temperature Regulation and Their Effect on the Catalytic Performance of Glycerol Steam Reforming for Hydrogen Production
Source: Nanomaterials (Basel). 2021 Nov 22;11(11):3149. doi: 10.3390/nano11113149 (PMC8620743; doi:10.3390/nano11113149)
Supplement: Supplementary file 1 [file nanomaterials-11-03149-s001.zip › nanomaterials-1441401-supplementary.pdf]

nanomaterials

MDPI

Supplementary Materials

Study of the Metal–Support Interaction and Electronic Effect Induced by Calcination Temperature Regulation and their Effect on the Catalytic Performance of Glycerol Steam Reforming for Hydrogen Production

Songshan Zhu [1,4,5], Yunzhu Wang [1,4,5], Jichang Lu [1,4,5], Huihui Lu [1,4,5], Sufang He[1,3,*], Di Song[1,4,5], Yongming Luo [1,2,4,5] and Jiangping Liu [1,4,5,*]

1 Faculty of environmental science and engineering, Kunming University of Science and Technology, Kunming 650500, China; Zhuss525@163.com (S.Z.); wyzkust@163.com (Y.W.); lujichangc7@kust.edu.cn (J.L.); luhuihuiluhuihui@163.com (H.L.); songdi0512@163.com (D.S.); environcatalysis@kust.edu.cn (Y.L.)
2 Faculty of chemical engineering, Kunming University of Science and Technology, Kunming 650500, China.
3 Research Center for Analysis and Measurement, Kunming University of Science and Technology, Kunming 650093, China.
4 The Innovation Team for Volatile Organic Compounds Pollutants Control and Resource Utilization of Yunnan Province, Kunming 650500, China.
5 The Higher Educational Key Laboratory for Odorous Volatile Organic Compounds Pollutants Control of Yunnan Province, Kunming 650500, China.
* Correspondence: shucai1983@163.com(S.H.); liujiangping@gig.ac.cn (J.L.); Tel: +86-871-65103845(J.L.).

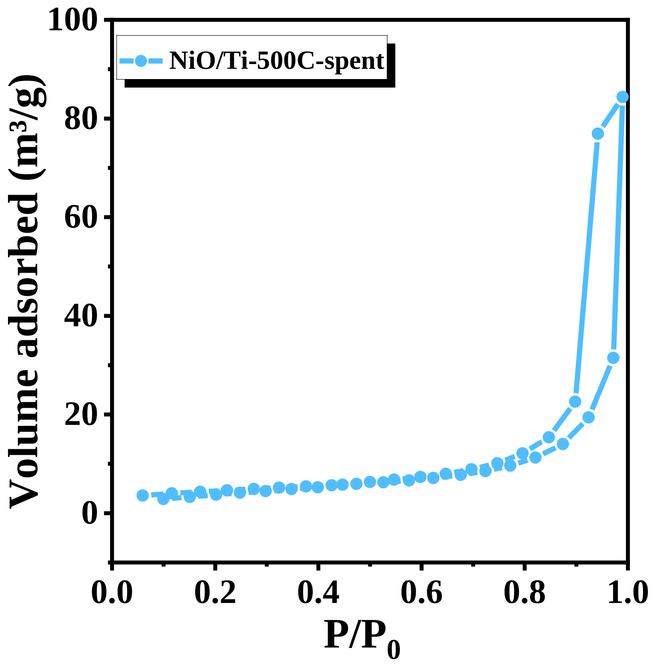

Figure S1. N_2 adsorption–desorption isotherms profile of the Ni/Ti-500R catalysts after 20h stability test.

The specific surface area, pore volume, and pore diameter are 19.60 m^3/g, 0.13cm^3/g and 12.48nm, respectively.

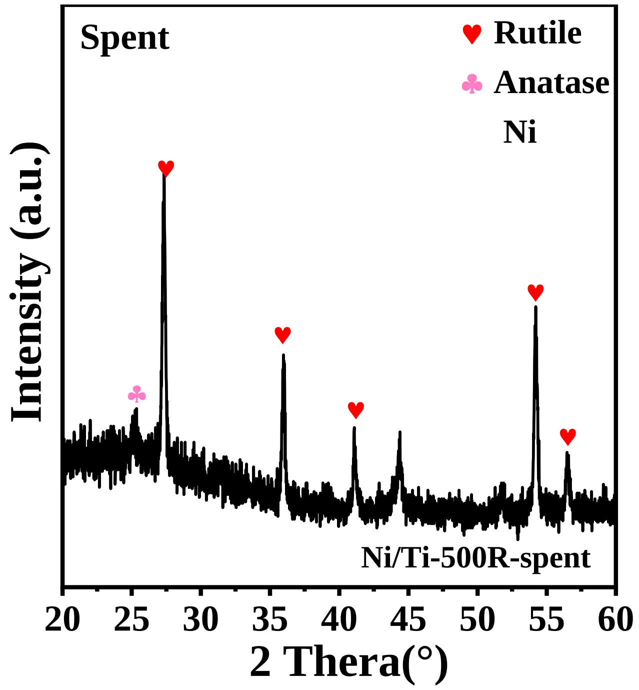

Figure S2. XRD pattern of the Ni/Ti-500R catalysts after 20 h stability test.

The particle size of Ni^0 for Ni/Ti-500R catalysts after 20 h stability test is to be 21.2 ± 0.5 nm. Compared with the reduced Ni0 particle size of Ni/Ti-500R, the particle size increases after high temperature reaction.
